# Generation of Tamm Plasmon Resonances for Light Confinement Applications in Narrowband Gradient-Index Filters Based on Nanoporous Anodic Alumina

**DOI:** 10.1021/acsanm.2c05356

**Published:** 2023-03-22

**Authors:** Alejandro Rojas Gómez, Laura K. Acosta, Josep Ferré-Borrull, Abel Santos, Lluis F. Marsal

**Affiliations:** † Department of Electronic, Electric, and Automatics Engineering, Rovira i Virgili University, Tarragona 43007, Spain; ‡ School of Chemical Engineering and Advanced Materials, The University of Adelaide, Adelaide, South Australia 5005, Australia; § Institute for Photonics and Advanced Sensing, The University of Adelaide, Adelaide, South Australia 5005, Australia

**Keywords:** nanoporous anodic alumina, gradient-index filters, Tamm plasmon resonance, photonic crystal, plasmonic−photonic hybrid structure

## Abstract

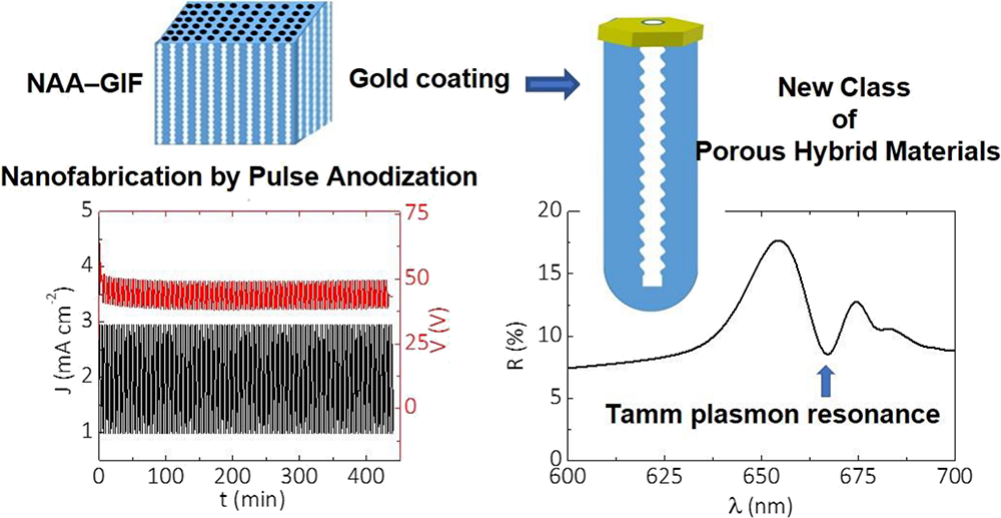

Gold-coated gradient-index filters based on nanoporous anodic alumina (Au-coated NAA–GIFs) were used as model platforms to elucidate how Tamm plasmons can be tailored by engineering the geometric features of the plasmonic and photonic components of these hybrid structures. NAA–GIFs with well-resolved, intense photonic stopbands at two positions of the visible spectrum were fabricated through sinusoidal pulse anodization. These model photonic crystals were used to assess how the quality of Tamm plasmon resonances can be enhanced by tuning the features of the dielectric mirror and the thickness of the porous gold coating layer. It is found that the highest value of the quality factor of Tamm resonance (*Q*_Tamm_ = 237) is obtained for 11 nm of gold on a dielectric mirror with low porosity corresponding to the resonant spectral position of λ_Tamm_ of ∼698 nm. Our analysis indicates that Tamm resonances in as-produced Au-coated NAA–GIFs are weak due to the constrained range of wavelengths (narrow bands) at which these photonic crystal structures reflect light. However, after broadening of their photonic stopband upon pore widening, Tamm resonances become better resolved, with higher intensity. It is also observed that the quality of light confinement worsens progressively with the thickness of the porous gold coating layer after a critical value. In contrast to conventional surface plasmon resonance systems, this hybrid Tamm porous system does not require complex coupling systems and provides a nanoporous structure that can be readily tailored for a range of photonic technologies such as sensing and lasing.

## 1 Introduction

Tamm plasmon resonances first proposed by Kaliteevski and coworkers are a class of surface plasmons in which incident light is confined at the interface of a hybrid structure consisting of a thin metallic coating layer (e.g., Au, Ag, and Pt) and a dielectric mirror (e.g., a Bragg reflector).^1^ Upon interaction with the plasmonic–photonic hybrid structure, electromagnetic waves induce oscillations of free electrons in the metal, which subsequently propagate across its surface and resonate at specific spectral regions within the interface between the thin metallic coating layer and the dielectric mirror.^2^ This constructive resonant confinement of light relies on the intrinsic optical properties of the plasmonic and photonic components of the hybrid structure (i.e., dielectric constants) and their geometric features (i.e., thickness and optical thickness, respectively).^3^ As such, this light–matter interaction can be precisely engineered across the broad optical spectrum by designing the properties and geometry of the plasmonic and photonic components of this type of hybrid optical structure.^1^

One of the most interesting aspects of Tamm plasmons is that resonant photons confined at and traveling along the plasmonic–photonic interface are highly susceptible to changes in the refractive index of the surrounding medium.^1−3^ For example, when molecules are immobilized onto the metal coating layer, the evanescent electromagnetic field associated with the flow of electrons along the surface of the metallic coating layer alters its properties due to the localized change in the refractive index. As such, the resonance band of the Tamm plasmon system undergoes a spectral shift, which is proportional to the change of the refractive index. This property in turn can be readily harnessed as the core principle to develop highly sensitive sensing systems.^4−7^

The sensitivity of Tamm plasmon systems depends on how strongly light is confined by the thin metallic coating layer–dielectric mirror composite, which can be quantified by the quality factor of the Tamm structure (*Q*_Tamm_)—the figure of merit defined as the ratio of the wavelength of the resonance band (λ_Tamm_) to its full width at half-maximum (FWHM_Tamm_). However, surface plasmon resonances are typically characterized by broad resonance bands due to the intrinsically high optical losses associated with the imaginary part of the dielectric constant of metals, which is associated with the energy losses experienced by photons when these interact with the atoms of the metal.^8,9^ This property can be beneficial for a range of photonic applications such as infrared imaging,^10,11^ solar energy conversion,^12^ and photocatalytic processes.^13,14^ However, other applications require a precise control over the spectral position and linewidth of surface plasmon resonances such as quantum optics,^15^ filtering,^16^ lensing,^17^ lasing,^18^ tweezing,^19^ and sensing.^20^

As such, plasmonic structures that can strongly confine light with high quality and narrow linewidths would provide new opportunities to develop advanced systems for emerging photonic technologies and applications. In this context, Tamm plasmons are a promising approach to generate narrowband plasmonic resonances since these can be excited at any angle of incidence for both polarizations without coupling prisms or gratings, which can be advantageous for integration and miniaturization.^9,10,21^

To date, a range of materials and structures have been engineered and integrated into Tamm plasmon systems. These include semiconductors,^22^ magnetophotonic crystals,^23^ and all-dielectric photonic crystals.^24^ Of all these, nanoporous dielectric photonic crystal (PC) structures are particularly suitable platforms for developing optical sensing systems that harness Tamm plasmons. This class of PCs provides a nanoporous matrix to increase available functional sites for maximizing binding interactions with targeted analyte molecules. Recently, nanoporous anodic alumina photonic crystals (NAA–PCs) produced by pulse-like anodization of aluminum^25−27^ have been demonstrated as suitable platforms to develop Tamm plasmon sensors.^1,25−27^

Conventionally, the photonic element of Tamm plasmon systems is based on a 1D distributed Bragg reflector (DBR) mirror, which is a PC structure featuring a periodic variation of optical thickness (i.e., the product of the refractive index and physical thickness) between high and low values in depth, along the mirror thickness.^28^ However, these PC structures are characterized by a broad photonic stopband, which in turn limits the quality of the resultant Tamm plasmon resonances.^29^ In contrast to DBRs, gradient-index filters (GIFs) feature a smooth, sinusoidal variation of optical thickness between high and low values, which is translated into a characteristically narrow photonic stopband (PSB) with suppressed side-lobe reflections around the central PSB.^30−32^

**Figure 1:**
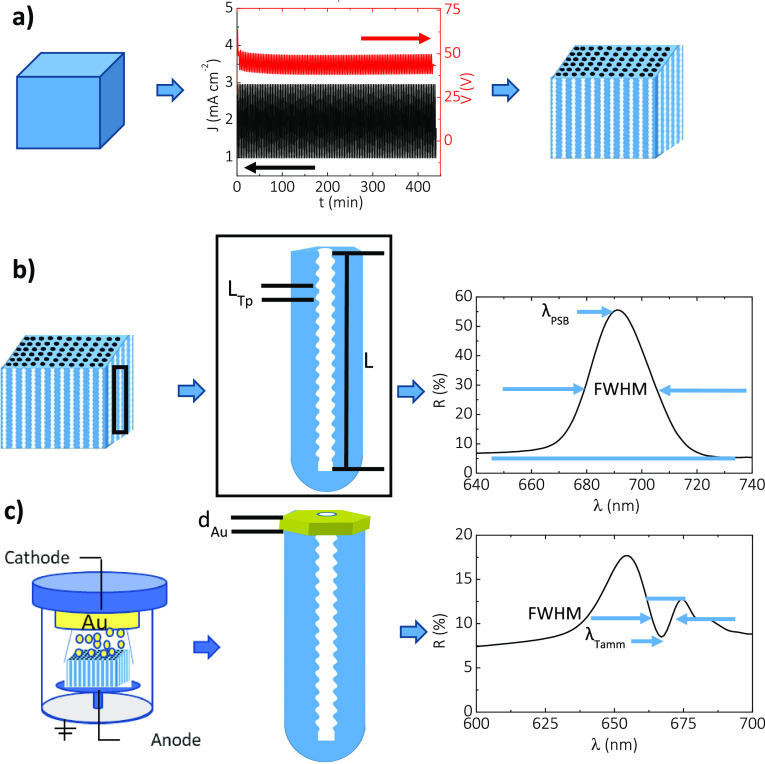
Concept of Tamm plasmon resonances in gold-coated nanoporous anodic alumina gradient-index filters (NAA–GIFs). (a) Electrochemical oxidation of aluminum substrates by a sinusoidal pulse-like anodization approach to generate NAA–GIFs. (b) Schematic illustrating the idealized nanoporous structure of NAA–GIFs featuring nanopore modulations in depth (left) and representative reflection spectrum of an as-produced NAA–GIF showing a well-resolved, intense, high-quality photonic stopband (right). (c) Illustration showing the coating process by a sputtering technique to generate a porous gold coating layer on top of NAA–GIFs (left) and the reflection spectrum of this structure showing a Tamm resonance within the visible region of the electromagnetic spectrum (NB: NAA–GIFs were coated, and a porous gold coating layer was obtained via sputter coating at 5 × 10^–5^ mbar).

In this study, we hypothesize that the quality of Tamm plasmon resonances can be enhanced by modifying the photonic stopband (PSB) of the dielectric mirror component of this hybrid plasmonic–photonic system. To this end, we engineer the structure of NAA–GIFs by sinusoidal pulse anodization^25,30,32−38^ (Figure 1a). These PC structures feature a well-resolved, intense, narrow photonic stopband in the visible spectrum, the spectral position of which can be precisely tuned by modification of the period in the input anodization profile (Figure 1b). Upon generation of a porous gold coating layer on top of the NAA–GIFs, these structures show a well-resolved, high-quality Tamm plasmon resonance at the center of their characteristic PSB (Figure 1c). We use these model platforms to elucidate how Tamm plasmons can be tailored by engineering the geometric features of the plasmonic and photonic components of these composite optical structures.

## 2 Experimental Section

### 2.1 Materials

High-purity aluminum (Al) sheets (i.e., 0.5 mm thickness and 99.99% purity) were acquired from Goodfellow Cambridge Ltd. (UK). Acetone ((CH_3_)_2_CO), ethanol (C_2_H_5_OH), perchloric acid (HClO_4_), oxalic acid (H_2_C_2_O_4_), hydrochloric acid (HCl), and copper chloride (CuCl_2_) were provided by Sigma-Aldrich (Spain). Double deionized water with a conductivity of 18 MΩ cm obtained by a dispensing system with an LC134 0.2 μm cutoff filter (ELGA LabWater) was used to prepare all aqueous solutions used in this study and to wash samples at the different stages of the fabrication process.

### 2.2 Fabrication of NAA–GIFs

Aluminum sheets were cut into squared chips with a side of 2 cm. The surface of as-received Al chips was cleaned sequentially with acetone, water, and ethanol to remove impurities before starting the fabrication process. The surface of cleaned Al chips was smoothened by electropolishing in a mixture electrolyte solution of 4:1 v/v ethanol–perchloric acid at 20 V and 5 °C for 8 min. During this process, the direction of stirring was alternated every 60 s. After electropolishing, Al chips were anodized by sinusoidal pulse anodization in a 0.3 M oxalic acid electrolyte at 5 °C. Current density sinusoidal anodization profiles were generated in a LabView customized application, following eq 1:
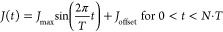
1where *J*(*t*) is the applied current density (in mA cm^–2^) at time *t* (in s), *J*_offset_ is the offset of the current density during the anodization process, *J*_max_ is the maximum current density in absolute value with respect to its average, *T* is the anodization period of the sinusoidal profile (in s), which represents the time difference between consecutive pulses, and *N* is the number of sinusoidal pulses in the anodization profile.

The values of *J*_offset_, *J*_max_, and *N* were fixed to 2 mA cm^–2^, 1 mA cm^–2^, and 120 pulses, respectively, whereas the input anodization period was set to two different values (i.e., *T* = 220 and 240 s, labeled as NAA–GIF–A and NAA–GIF–B, respectively) to produce NAA–GIFs with PSBs located at two specific positions of the visible region of the electromagnetic spectrum.

### 2.3 Structural Characterization of NAA–GIFs

The structure of NAA–GIFs was characterized by a field-emission gun scanning electron microscope (FEG–SEM, Thermo Fisher Scientific model Scios 2) operating at an accelerating voltage of 5 kV. FEG–SEM images were analyzed by ImageJ software to quantify the average geometric features of NAA–GIFs.^39^ After fabrication, NAA–GIFs were coated, and a porous gold coating layer was obtained through a sputtering technique in a Quorum Q 150T Plus turbomolecular pumped coater in the Q 150T S Plus configuration at a vacuum pressure of 5 × 10^–5^ mbar. These conditions make it possible to obtain a low grain size of the metal coating layer to minimize undesired scattering, which is a suitable characteristic to study the optical properties of plasmonic–photonic interactions at the interface of metal/NAA–GIFs. The average thickness of the resultant porous gold coating layer was estimated using a KLA-Tencor Alpha-Step D-300 stylus profiler. A porous gold coating layer generated in this study had an average thickness from 6 ± 1 to 23 ± 1 nm. Quantification of porous gold coating layer thickness by this technique was calibrated by partially masking the surface of reference samples during the coating process. Then, the thickness of the resultant porous gold coating layer was determined by scanning the surface of the sample with the stylus profiler across the transition between masked and unmasked regions. The height of the step corresponds to the average thickness of the porous gold coating layer.

### 2.4 Optical Characterization of NAA–GIFs

The aluminum substrate remaining after anodization was removed from the bottom side of the NAA–GIFs by selective chemical etching in a saturated solution of CuCl_2_·2H_2_O 0.16 M dissolved in a 1:4 v/v mixture of HCl and H_2_O at room temperature, using a circular mask to create a transparent window for optical characterization purposes.^32^ The absolute reflectance spectra (expressed in %*R*) of NAA–GIFs were acquired in a PerkinElmer UV–visible–NIR Lambda 950 spectrophotometer within the visible range (i.e., 450–800 nm), measured with a 1 nm resolution at an 8° incidence angle.

## 3 Results and Discussion

### 3.1 Structural Characterization of NAA–GIFs

Periodic sinusoidal variation of the anodizing current density input enables a precise means of modulating the effective medium of NAA by generating regions of higher and lower porosity along the nanopores (Figure S1a). These regions in turn correspond to periodic variations of low and high levels of the effective refractive index, respectively. From magnified views of the anodization profile, it is apparent that there is a delay of 40 ± 20 s between the current density input and the voltage output profile (Figure S1b,c).

This is attributed to the restructuring of the barrier oxide layer at the bottom of the nanopores (i.e., anodic film growth front) when the input current density changes from high to low values. It is worth noting that this anodization process is entirely performed under mild conditions and that, as such, there is a slight decrement in the average voltage (i.e., (*V*_max_*–V*_min_)/2) with the anodization time. This process results in the modulation of the nanopore diameter from top to bottom of the anodic film by the variation of input current density in the form of pulses with a smooth transition from high to low values.^40^ This in turn generates alternating zones of low and high effective refractive index.^41^

The smooth variation of the effective refractive index induced by the sinusoidal pulse anodization profile results in the generation of a gradient-index filter photonic crystal structure (NAA–GIFs), which is characterized by a well-resolved, narrow photonic stopband (PSB) in the reflection spectrum.

**Figure 2:**
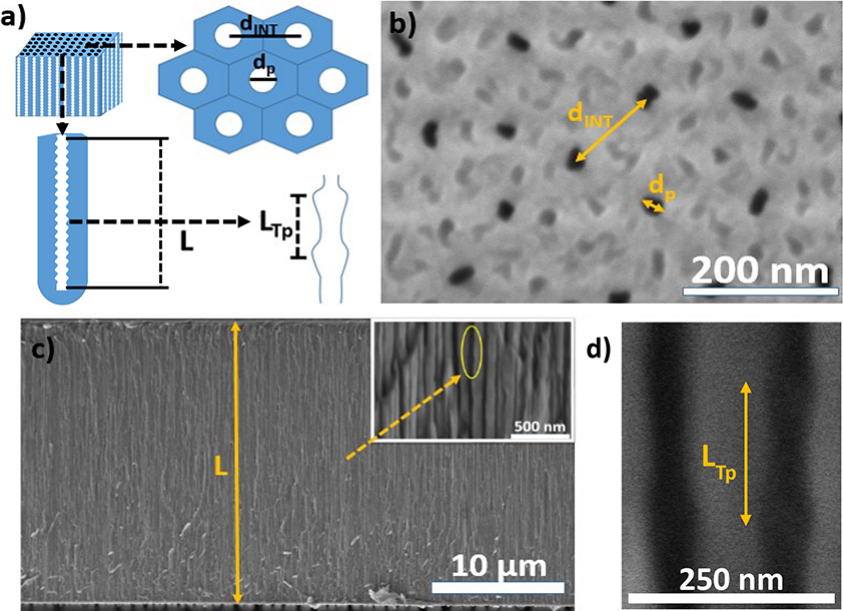
Structural characterization of NAA–GIF–A produced by sinusoidal pulse anodization. (a) Schematic representation of structural parameters obtained after fabrication with a brief description of the top view and nanopore modulation in depth. In all cases, the structural parameters are graphically defined: *d*_p_, pore diameter; *d*_int_, interpore distance; *L*, NAA–GIF thickness; *L*_TP_, periodic modulation thickness. (b) Top-view image of the random pore distribution of fabricated NAA–GIFs showing nanopores with an average diameter *d*_p_ and interpore distance *d*_int_ of 23 ± 4 and 93 ± 21 nm, respectively; (c) general cross-sectional view of representative NAA–GIFs showing nanopores with an overall thickness *L* of ∼23 μm. In the upper right inset, it shows circled in yellow a magnified cross-sectional view of fabricated NAA–GIFs; pore modulation during the fabrication process (d) where a magnified cross-sectional view of fabricated NAA–GIF reveals the modulated porosity along the length with the period length or distance *L*_Tp_ between nanopore modulations of 194 ± 17 nm.

Figure 2a shows a top-view FEG–SEM image of a representative as-produced NAA–GIF–A fabricated with an anodization period of *T* = 220 s. This structure is characterized by a random distribution of nanopores across the photonic crystal film surface, the average diameter (*d*_p_) and the interpore distance (*d*_int_) of which are estimated to be 23 ± 4 and 93 ± 21 nm, respectively.^30,32^Figure 2b,c shows general and magnified cross-sectional-view images of the as-produced NAA–GIF–A structure. Under the conditions of fabrication used in our study, the photonic films feature an overall thickness (*L*) of ∼23 μm. A magnified-view image of the cross section reveals periodic modulations of the diameter along the nanopores, which go from the top to the bottom of the anodic film (Figure 2c).

It is apparent that the sinusoidal modulation of current density input in the anodization profile is precisely translated into sinusoidal variations of the nanopore diameter. The period length (*L*_Tp_) or distance between nanopore modulations estimated by FEG–SEM image analysis was determined to be ∼194 ± 17 nm. Similar results are obtained for sample NAA–GIF–B, manufactured with an anodization period of *T* = 240 s, which translates into an overall thickness (*L*) of ∼26 μm with the same average diameter (*d*_p_) and interpore distance (*d*_int_), but the distance between nanopore modulations estimated by FEGSEM image analysis was determined to be ∼202 ± 11 nm.

### 3.2 Optical Characterization of NAA–GIFs

PCs are optical nanostructures with a spatial periodic variation of the dielectric constant in one, two, or three dimensions, which determines the way in which they interact with incident photons. NAA has emerged as a promising platform material for a range of PC structures that enable a versatile control over a range of light–matter interactions (e.g., light confinement and recirculation,^42^ selective filtering and reflection,^43^ slow light,^44^ and light emission^45^). Of all these, NAA–GIFs are characterized by a smooth periodic variation of the dielectric constant along the nanopore length. This structural feature is translated into a characteristically narrow PSB in the reflection spectrum of these PC structures, which indicates a highly selective inhibition of the flow of photons within a highly constrained range of energies or wavelengths. Furthermore, since NAA–PCs are a porous material, the macroscopic optical properties of these structures can be mechanistically described by effective medium approximation.^38,46−48^

In this approach, the property of each constituent phase (air and alumina) is averaged according to the spatial distribution of the photonic crystal architecture (i.e., gradient-index filter in this case) as well as the contribution of each phase separately (i.e., its own dielectric constant or refractive index).^49^ In this way, by engineering the structure of the NAA–GIFs and modulating its nanopores through sinusoidal anodization, we can tailor the characteristic spectroscopic fingerprint of this material to harness light–matter interactions at the nanoscale though induced changes of the effective refractive index in depth. In the case of NAA–GIFs, the flow of incoming light is strongly forbidden within a narrow region of the electromagnetic spectrum, which translated into a narrow PSB (i.e., the optical range within which light is strongly reflected by the PC structure).

**Figure 3:**
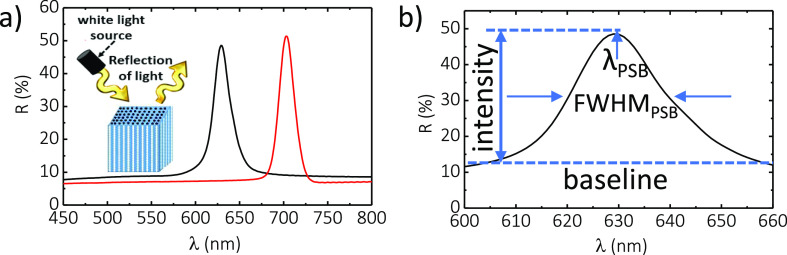
Optical characterization of NAA–GIFs. (a) Reflectance spectra (expressed in %*R*) of the NAA–GIF corresponding to samples NAA–GIF–A (black line) and NAA–GIF–B (red line) and a schematic diagram of light reflection. (b) Definition of main optical features of the PSB of these PCs in their reflection spectra (blue dashed line: baseline): central wavelength (λ_PSB_), full width at half-maximum FWHM_PSB_, and the intensity of the stopband, *I*_PSB_. The PSBs of NAA–GIF–A and NAA–GIF–B fabricated with anodization periods of *T* = 220 and 240 s have central wavelengths of 632 ± 1 and 704 ± 1 nm, with full widths at half-maximum of 22 ± 1 and 21 ± 1 nm, respectively.

Figure 3 shows the characteristic reflection spectrum of NAA–GIFs type A and B, where both PC structures show well-resolved, intense PSBs in the upper region of the visible spectrum (i.e., from ∼600 to ∼750 nm). Both NAA–GIFs were fabricated under the same conditions but under distinct anodization periods (*T* = 220 and 240 s for A and B, respectively). It is apparent from these spectra that the position of the PSB of NAA–GIFs red-shift with the anodization period, enabling a precise means of engineering this light–matter interaction across the broad spectrum through this anodization parameter. The PSBs of NAA–GIF–A and NAA–GIF–B fabricated with anodization periods of *T* = 220 and 240 s have their central wavelength (λ_PSB_) at 632 ± 1 and 704 ± 1 nm, with full widths at half-maximum (FWHM_PSB_) of 22 ± 1 and 21 ± 1 nm, respectively.

### 3.3 Influence of Pore Widening on Optical Properties of NAA–GIFs

Selective reflection of light in NAA–GIFs is attributed to multiple Bragg scattering interactions across the NAA stacks featuring a sinusoidally modulated nanopore diameter along the thickness of the anodic film. The central wavelength of the characteristic PSB of NAA–GIFs indicates the spectral region where the nanoporous PC forbids the flow of incoming photons more efficiently due to constructive interference. This light–matter interaction can be controlled by engineering the period length (i.e., thickness of NAA stacks forming the NAA–GIF structure) and the effective refractive index of the PC structure. Whereas the former can be precisely controlled by the period in the anodizing current density input, the latter relies intrinsically on porosity or the nanopore diameter.

**Figure 4:**
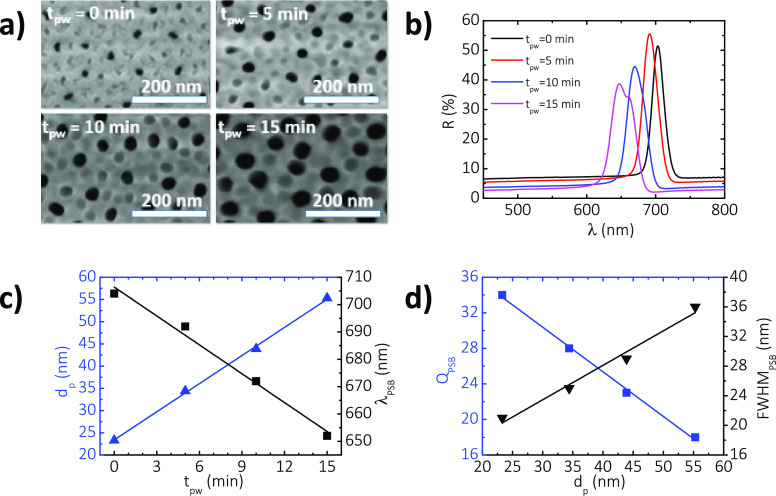
Analysis of the effect of the nanopore diameter on NAA–GIFs. (a) Effect of the pore widening time (*t*_pw_ = 0, 5, 10, and 15 min) on the geometric and optical properties of NAA–GIFs (i.e., NAA–GIF–B). By increasing the pore widening time, an enlargement of the nanopores on the top of the sample surface occurs, from 21 ± 1 to 36 ± 1 nm. (b) Representative reflection spectrum of NAA–GIF–B as a function of *t*_pw_ from 0 to 15 min. The position of the photonic stopband blueshifts when the pore widening time increases and the FWHM_PSB_ becomes wider. (c) Linear fitting correlating the growth in the average pore diameter with increasing pore widening time (solid blue line) and linear fitting line showing the blue-shift between λ_PSB_ and *t*_pw_ (solid black line). (d) Linear fitting line showing the dependence between FWHM_PSB_ and the quality factor *Q*_PSB_ on *d*_pw_. As the porosity of the sample increases, the photonic stopband becomes wider and the quality factor decreases.

To assess the effect of the average nanopore diameter on the optical properties of NAA–GIFs, this geometric feature was modified by wet chemical etching over controlled pore widening times (*t*_pw_) of 0, 5, 10, and 15 min. FEGSEM image analysis determined average nanopore diameters (*d*_p_) from the top surface of the anodic photonic film of 23 ± 4, 34 ± 4, 44 ± 5, and 55 ± 5 nm for these values of *t*_pw_, respectively (Figure 4a). It is apparent that the position of the PSB of NAA–GIF–B undergoes a spectral blue-shift with increasing average nanopore diameter (Figure 4b). The central wavelengths of these NAA–GIFs were determined to be located at λ_PSB_ = 704 ± 1, 692 ± 1, 672 ± 1, and 652 ± 1 nm after 0, 5, 10, and 15 min of pore widening treatment, respectively.

It is possible to distinguish two zones with different reflectance intensity behaviors as the porosity of the NAA–GIF–B structure increases: initially, %*R* increases with porosity up to reaching a maximum when the nanopore diameter becomes ∼32 nm. After this point, %*R* decreases progressively with *t*_pw_ until reaching its minimum at an ∼55 nm nanopore diameter. This behavior is in good agreement with previous observations and has been attributed to diffusive light scattering by the photonic crystal structure due to an increase in its porosity.^30^ At low porosity, light is scattered less efficiently by the PC structure. However, as the average nanopore diameter increases, electromagnetic waves lose energy more efficiently as they travel across the photonic structure.

The dependence of the average *d*_p_ on *t*_pw_ is shown in Figure 4c, which reveals a strongly linear correlation between these two parameters (*R*^2^ = 0.9989) with a nanopore enlargement of 2.1 ± 0.1 nm min^–1^ with *t*_pw_. Figure 4c also reveals a linear correlation (*R*^2^ = 0.9878) between the position of the central wavelength and *t*_pw_, in which the latter optical feature blueshifts at a rate of 3.5 ± 0.3 nm min^–1^. This result is consistent with previously reported works^28−32,38,50,51^ and is attributable to the reduction of the overall effective refractive index of the photonic film with increasing porosity, where the fraction of air (lower refractive index) increases over that of alumina (component of the composite structure with a higher refractive index).

If we correlate the dependence of λ_PSB_ with *d*_p_ for NAA–GIFs, it reveals a linear correlation (*R*^2^ = 0.9849) where the position of the central wavelength blueshifts at a rate of 1.7 ± 0.1 nm nm^–1^ with *d*_p_ (further information can be found in Figure S2 of the Supporting Information). Therefore, it is possible to control the position of the PSB with high precision by adjusting the size of the nanopore in these PC structures, providing an additional degree of freedom to fine-tune the optical features of the PSB. This analysis also indicates that the characteristic PSB of NAA–GIFs broadens upon enlargement of the nanopore diameter through pore widening (Figure 4d). Analysis of the dependence of the full width at half-maximum (FWHM_PSB_) with pore widening time reveals a linear broadening of the band (*R*^2^ = 0.9826), with values of 21 ± 1, 25 ± 1, 29 ± 1, and 36 ± 1 nm at *t*_pw_ of 0, 5, 10, and 15 min, respectively (i.e., a total increment of ∼14 nm at a change rate of 0.5 ± 0.1 nm nm^–1^ with *d*_p_).

Also, we define in eq 2 a way to calculate the quality factor *Q*_PSB_ of the photonic stopband (PSB) based on the relationship between the central wavelength and its FWHM. The behavior of this figure of merit when increasing the average pore diameter is also represented in Figure 4d in a solid blue line. This behavior reveals a linear decreasing of *Q*_PSB_ (*R*^2^ = 0.9974), with values of 34 ± 1, 28 ± 1, 23 ± 1, and 18 ± 1 at *d*_p_ of 23 ± 4, 34 ± 4, 44 ± 5, and 55 ± 5 nm, respectively (i.e., a total reduction of ∼16 units at a change rate of −0.5 ± 0.1 units nm^–1^ with *d*_p_). Table S1 in the Supporting Information summarizes the values of the different optical and geometric parameters at distinct pore widening times.
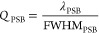
2

### 3.4 Influence of Gold Coating on Structural and Optical Properties of NAA–GIFs

Tamm plasmons are a class of light–matter interactions in which incoming photons are confined between a metallic coating layer and a dielectric mirror. In this system, the geometric and optical features of the metallic coating layer are critical in determining the capability of this type of light-confining hybrid plasmonic–photonic structure. To evaluate the influence of the thickness of the metallic coating layer on the optical features of Tamm plasmons in NAA–GIFs, layers of gold with controlled thicknesses were deposited on the top surface of these NAA-based PC structures by a sputtering technique. Four thicknesses of the gold coating layers were deposited on the surface of the NAA–GIF–A and NAA–GIF–B structures by controlling the sputtering time (Figure S2 in the Supporting Information).

Preliminary calibration experiments revealed gold coating layer thicknesses of 6 ± 1, 8 ± 1, 11 ± 1, and 23 ± 1 nm at 10, 20, 30, and 60 s of deposition time under the conditions used in our system, respectively. Figure S4 shows top-surface FEGSEM images of a representative NAA–GIF–A after 15 min of pore widening and at different thicknesses of gold coating (i.e., from 6 to 23 nm). Even after pore widening, there is a quantifiable reduction in the pore diameter with increasing thickness of gold coating. The results summarized in Table S2 reveal that coating on top of the sample leads to the formation of a 23 ± 1 nm porous gold coating layer that reduces the pore diameter size of the sample itself.

**Figure 5:**
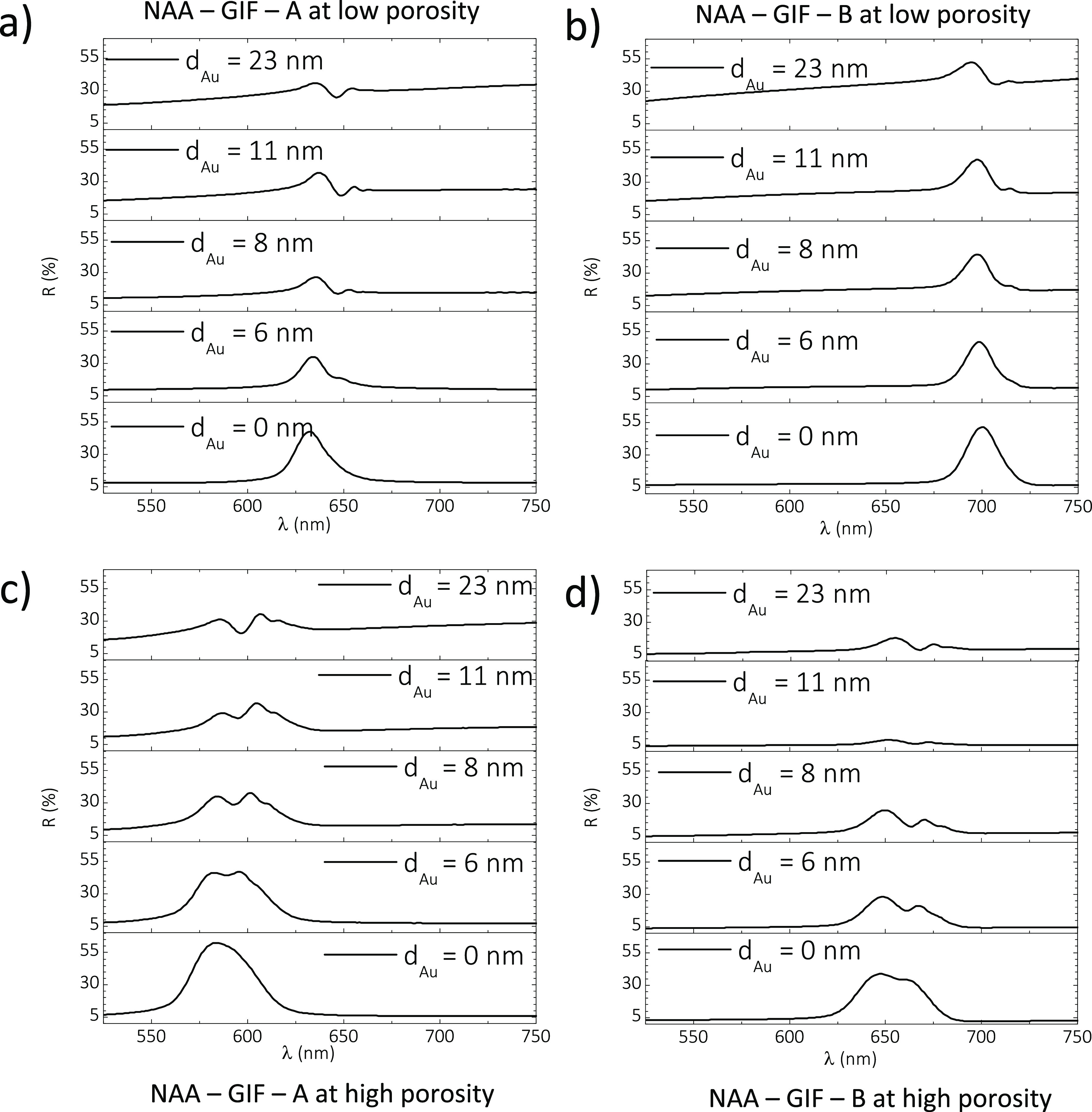
Analysis of the effect of gold coating on the reflectance spectrum of as-produced NAA–GIFs (low porosity and high porosity). (a) Reflection spectra of as-produced NAA–GIF–A coated with different thicknesses of gold at low porosity. (b) Reflection spectra of as-produced NAA–GIF–B coated with different thicknesses of gold at low porosity. (c) Reflection spectra of as-produced NAA–GIF–A coated with different thicknesses of gold at high porosity. (d) Reflection spectra of as-produced NAA–GIF–B coated with different thicknesses of gold at high porosity.

Analysis of the reflection spectrum of as-produced Au-coated NAA–GIFs reveals an apparent dip within the PSB in the original spectrum, which is attributed to the coupling between the plasmonic and photonic modes at the physical interface between the porous gold coating layer and the dielectric mirror^2^ (Figure 5,ba). For the thinnest coating (i.e., 6 nm), the dip in the reflectance spectrum is barely resolved in both NAA–GIF–A and NAA–GIF–B, but this resonance band is more marked for NAA–GIF–A, which features a PSB located at shorter wavelengths (i.e., λ_PSB_ = 632 ± 1 nm) (Figure 5a). A qualitative enhancement in resolution of the Tamm plasmon resonance occurs when the thickness of the porous gold coating layer is increased from 6 to 8 nm of sputtered gold. This enhancement is accompanied by an increase in the baseline at thicker thicknesses (i.e., 11 and 23 nm), which can be attributed to an overall increment in reflection due to the increase in density in the porous gold coating layer.

**Figure 6:**
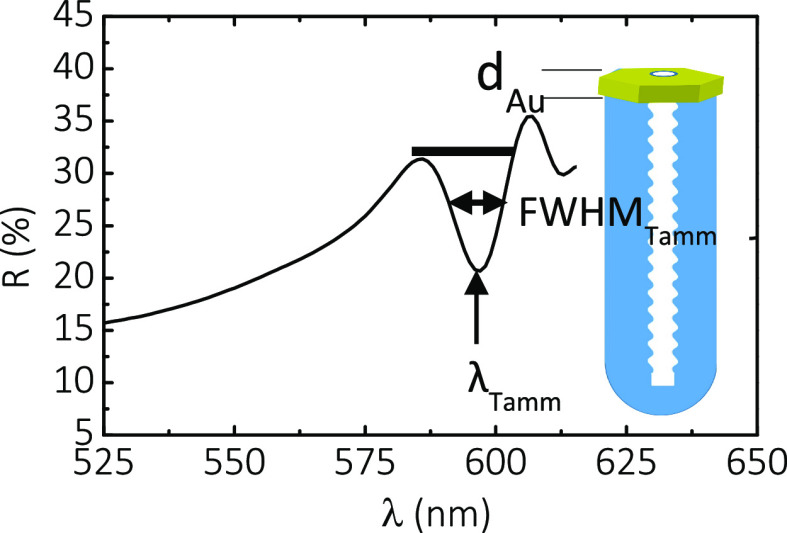
Illustration describing the spectral and structural parameters of Au-coated NAA–GIFs. The solid black line represents the characteristic reflection spectrum of Au-coated NAA–GIFs. This spectrum shows a well-resolved resonance band at approximately its center, which denotes the Tamm resonance λ_Tamm_*t*. At the right side of the spectrum, the increase in the thickness of the gold layer (yellow) coated on top of a GIF (blue and white) with sinusoidal modulated nanopores is presented.

The figure of merit defining how good a Tamm plasmon cavity confines light within its structure is the quality factor, defined graphically in Figure 6 and estimated quantitatively by eq 3 as follows:
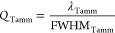
3where *Q*_Tamm_ is the quality factor of the central wavelength (λ_Tamm_) based on the relationship between the central wavelength and its FWHM_Tamm_.

At thin thicknesses of gold coating, the Tamm resonance is poorly resolved: we cannot calculate FWHM_Tamm_ for porous gold coating layer thicknesses *d*_Au_ of 6 and 8 nm. However, it becomes better resolved with *d*_Au_, and the increase of FWHM_Tamm_ becomes clearer for sample NAA–GIF–A: FWHM_Tamm_ broadens with values of 10 ± 1, 13 ± 1, 15 ± 1, and 16 ± 1 nm at *d*_Au_ of 6 ± 1, 8 ± 1, 11 ± 1, and 23 ± 1 nm, respectively (i.e., a total increment of ∼6 nm with *d*_Au_). For the last two values of *d*_Au_*,* 11 ± 1 and 23 ± 1 nm, sample NAA–GIF–B is narrower (FWHM_Tamm_ measures 3 ± 1 and 5 ± 1 nm, respectively), and according to the figure of merit defined in eq 3, we can obtain higher quality factors *Q*_Tamm_, although the tendency in both samples is to decrease *Q*_Tamm_ with the increase in *d*_Au_.

Motivated by this result, we decided to assess the combined effect of a porous gold coating layer thickness and the average nanopore diameter for NAA–GIFs. The pore size of NAA–GIFs was increased via pore widening. After 15 min of pore widening, the PSB of NAA–GIF–A and NAA–GIF–B undergoes an ∼45 nm blueshift with a concomitant broadening of the average FWHM, which is estimated to be ∼14 nm. It is apparent that the pore widening treatment makes the PC structure evolve from a GIF (narrow band) to a distributed Bragg reflector, which is characterized by a broad PSB denoting an efficient reflection of light across a wider range of wavelengths within the visible spectrum. As a result, the Tamm resonance λ_Tamm_, represented by the dip in reflectance, can be appreciated more clearly within the PSB of the PC structure as shown in the reflection spectra depicted in Figure 5c,d.

It is apparent from Figure 5c,d that, upon a pore widening treatment of 15 min, the resolution of Tamm plasmon resonances within the characteristic PSB of NAA–GIF–A and NAA–GIF–B is enhanced at any thickness of the porous gold coating layer, from 6 to 23 nm. It is also found that the position of the Tamm plasmon resonance (λ_Tamm_) in the reflection spectrum of NAA–GIFs red-shifts with the porous gold coating layer thickness (*d*_Au_). These graphs reveal that the intensity of the Tamm resonance increases with the porous gold coating layer thickness, and this effect is more clearly noticeable for NAA–GIFs with PSBs located at shorter wavelengths (i.e., NAA–GIF–A).

**Figure 7:**
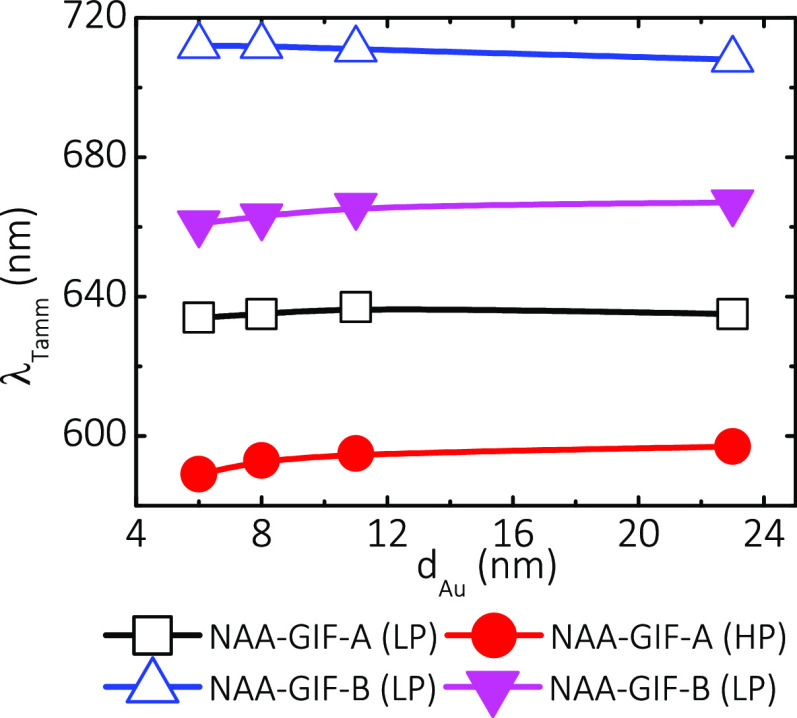
Evolution of λ_Tamm_ with increasing gold thickness (*d*_Au_). NAA–GIF–A shows that the position of the resonant signal λ_Tamm_ increases with *d*_Au_ at LP up to the value *d*_Au_ = 11 nm, and then, it starts to decrease. This limit point widens for higher porosities (HP), and the sample allows coatings of higher *d*_Au_ without a decrease in the signals λ_Tamm_. The same behavior is observed for sample NAA–GIF–B, but what happens at larger wavelengths is clearer at relatively high porosities (HP) because GIF nanopores are covered more easily at LP and the reflection spectrum begins to distort very soon.

To gain further insights, we extended this qualitative analysis by quantifying how the optical features of the Tamm plasmon resonance in these NAA–GIFs structures change with the levels of porosity induced by the pore widening time (i.e., low porosity, LP: as-produced; high porosity, HP: after 15 min of pore widening) (Figure 7). It can be noted that for LP, λ_Tamm_ redshifts its position with increasing gold thickness until reaching a critical point at *d*_Au_ = 11 nm, from which λ_Tamm_ blueshifts its position. In contrast, for the HP range, λ_Tamm_ redshifts its position asymptotically with the porous gold coating layer thickness. A potential reason for this phenomenon is the homogeneity of the porous gold coating layer. As-produced NAA–GIFs have more available surface to grow a solid, continuous layer of gold as the thickness of the deposited layer increases. Conversely, after an extended pore widening treatment, nanopores are larger, and as such, the available surface on top of the NAA–GIFs that is available for coating is substantially reduced.

**Figure 8:**
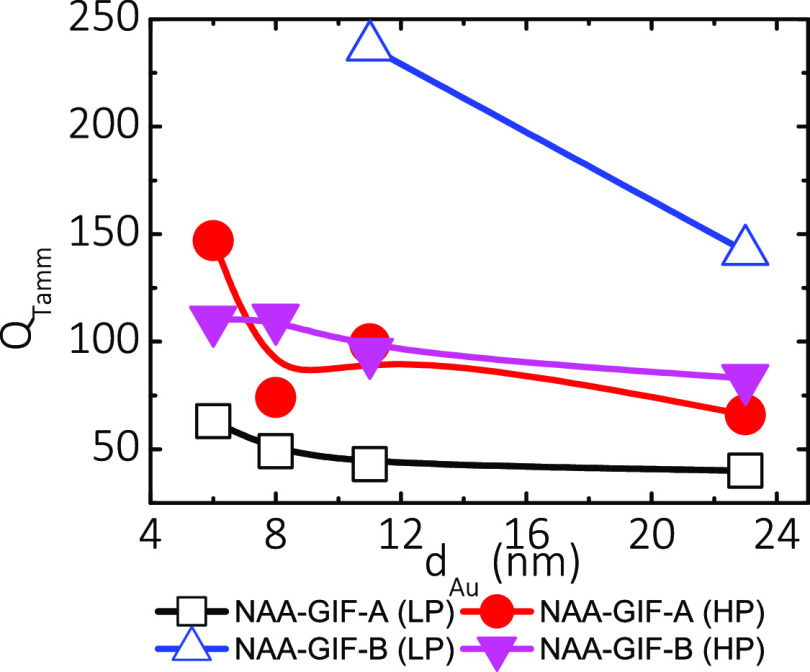
Influence of gold coating on NAA–GIFs at different wavelengths and porosity levels on the quality factor of the resonant signal. There is an increase in quality factors of the Tamm resonance signal *Q*_Tamm_ at high porosities (HP) and shorter wavelengths (sample NAA–GIF–A) for each value of *d*_Au_. The highest values of the *Q* factor are found at low porosities and at longer wavelengths (sample NAA–GIF–B). The largest absolute value is found for 11 nm gold thickness (*d*_Au_ = 11 nm). In both samples, we should expect a decrease in *Q*_Tamm_ as the amount of gold coated (*d*_Au_) on the samples increases, governed by the existing balance between λ_Tamm_ and FWHM_Tamm_, according to eq 2. The resonant signal broadens faster than its wavelength changes. See Table S2 in the Supporting Information.

Figure 8 shows the effect of the gold coating on the *Q*_Tamm_ for NAA–GIF–A and NAA–GIF–B at *t*_pw_ = 0 and 15 min (i.e., LP and HP, respectively). The highest value of the quality of light confinement (*Q*_Tamm_ = 237) is obtained at 11 nm of gold thickness for as-produced NAA–GIF–B. For NAA–GIF–A, the average *Q*_Tamm_ fluctuates around 49 ± 10 before pore widening (LP), and it is enhanced by almost two-fold to 97 ± 37 after 15 min of pore widening (HP). Although λ_Tamm_ decreases due to pore widening, the Tamm resonance is resolved much more for HP than for LP due to a narrower FWHM_Tamm_, and the resulting effect is an increase in *Q*_Tamm_. NAA–GIF–B shows a very similar trend in terms of *Q*_Tamm_, but we can only calculate it for the highest values of *d*_Au_ due to the poor resolution of the resonant signal for coatings thinner than 11 nm. The average *Q*_Tamm_ is estimated to be 190 ± 67 at LP and 100 ± 13 after a 15 min pore widening treatment. In all the cases, *Q*_Tamm_ decreases after the initial coating. However, *Q*_Tamm_ decreases more sharply in the case of NAA–GIF–B for LP and in the case of NAA–GIF–A for HP.

To compare the Tamm resonances of the hybrid structure with the photonic stopband of the GIFs, we analyzed how the quality factor *Q*_Tamm_ changes with the gold thickness *d*_Au_ before and after pore widening (Figure 8). The reference in this comparison is sample NAA–GIF–B, which showed the highest value of *Q*_PSB_ (*Q*_PSB_ = 34 ± 1), without pore widening; see Figure 4d. By engineering the nanostructure of the GIFs through pore widening and combining it with gold coating, we have been able to create different hybrid structures with series of higher *Q* values than the aforementioned reference in all cases (Table S2). This result, in principle, justifies the use of the novel hybrid structures based on Au-coated NAA–GIFs for future sensing and lasing experiments. Of all the gold thicknesses studied, the highest *Q*_Tamm_ (*Q*_Tamm_ = 237 ± 1) is obtained for 11 nm of gold on a sample of relatively low porosity (as-produced) corresponding to the resonant signal λ_Tamm_ of the NAA–GIF–B sample.

Our observations indicate that Tamm resonances in Au-coated NAA–GIFs are weak due to the constrained range of wavelengths (narrow bands) at which these PC structures reflect light. The selected fabrication parameters (current densities and anodization period) define, in principle, the reflectance levels of the NAA. The photonic stopbands show around 50% reflectance, which, despite having sufficient sensitivity (relatively narrow according to its FWHM) even for applications based on the angular response, constitutes a limitation for obtaining strong Tamm plasmons.^52^ However, after pore widening of the NAA–GIFs and gold coating, Tamm resonances become more resolved, with a higher intensity.

## 4 Conclusions

In summary, this work demonstrates that a sinusoidal pulse-like anodization approach in combination with sputtering makes the generation of Au-coated NAA–GIFs as a model hybrid metal–dielectric platform with a narrow photonic stopband and Tamm plasmon resonances within specific ranges of the visible electromagnetic spectrum possible. The spectral position of the photonic stopband of model NAA–GIFs was tuned by modifying the anodization period in the input profile.

After fabrication, we further engineered the features of the characteristic PSB of NAA–GIFs by pore widening, where this treatment makes it possible to broaden the band and blueshift its position. Then, model NAA–GIFs (dielectric) were combined by a sputtering technique to generate a porous gold coating layer on the top surface of these PCs. Upon coating, Tamm plasmon resonances were observed in the reflectance spectra of these structures. We then studied the effect of the gold coating on the *Q* factor of each optical signal at two levels of porosity and at two regions of the visible spectrum.

As-produced NAA-based GIFs feature Tamm resonances after coating their top surface with noncontinuous gold layers. However, the quality of light confinement worsens progressively with the porous gold coating layer thickness after a critical value. This constraint can be addressed by widening the nanoporous structure and broadening its characteristic photonic stopband. The highest value of the *Q* factor is obtained for 11 nm of gold on a sample of relatively low porosity (as-produced) corresponding to the resonant signal λ_Tamm_ of the NAA–GIF–B sample (at a relatively longer wavelength in the visible range of the electromagnetic spectrum) although a decrease in the *Q* factor can be expected as the amount of gold deposited increases_._

Very few theoretical investigations have focused on the use of metal in contact with GIFs at the contact interface.^35−37^ Although Tamm plasmon modes were predicted years ago, empirical demonstrations of this phenomenon are recent, and it is still an area of development. The main advantage of Tamm plasmon systems is that, in contrast to conventional SPR systems that require the coupling with a prism and complex optics, these are simple since they do not need any specific arrangement or complex optics. Also, they have the advantage that they can be used with white light illumination and can be engineered to work across the spectrum.

Our advances provide new opportunities to develop novel nanostructures in the development of new sensing or biosensing platforms. The optical properties of the combined material, the propagation of the electromagnetic field through the physical interface of separation, and its sensing capacity, among other issues, continue to be challenges for nanoplasmonics that is in full development.

## Supporting Information Available

Further information on estimation of structural and optical parameters of NAA–GIFs for different pore widening times, pore diameter rates, gold thickness rates according to a determined coating time, overlapped reflectance spectra of Au-coated NAA–GIFs with/without pore widening, optical features of Au-coated NAA–GIFs, and pore diameter estimation of Au-coated NAA–GIFs after gold sputtering (PDF)
