# Supplementary material for: Generation of Tamm Plasmon Resonances for Light Confinement Applications in Narrowband Gradient-Index Filters Based on Nanoporous Anodic Alumina
Source: ACS Appl Nano Mater. 2023 Mar 22;6(7):5274–83. doi: 10.1021/acsanm.2c05356 (PMC10112486; doi:10.1021/acsanm.2c05356)
Supplement: Supplementary file 1 — an2c05356_si_001.pdf [file an2c05356_si_001.pdf]

Supporting Information

Generation of Tamm Plasmon Resonances for Light Confinement Applications in Narrowband Gradient-Index Filters Based on Nanoporous Anodic Alumina

Alejandro Rojas Gómez[1], Laura K. Acosta[1], Josep Ferré-Borrull[1], Abel Santos[*2,3] and Lluis F. Marsal[*1]

[1]Department of Electronic, Electric, and Automatics Engineering, Rovira i Virgili University, Tarragona 43007, Spain
[2]School of Chemical Engineering and Advanced Materials, The University of Adelaide, South Australia 5005, Australia
[3]Institute for Photonics and Advanced Sensing, The University of Adelaide, South Australia 5005, Australia

[*]E-mails: abel.santos@adelaide.edu.au ; lluis.marsal@urv.cat

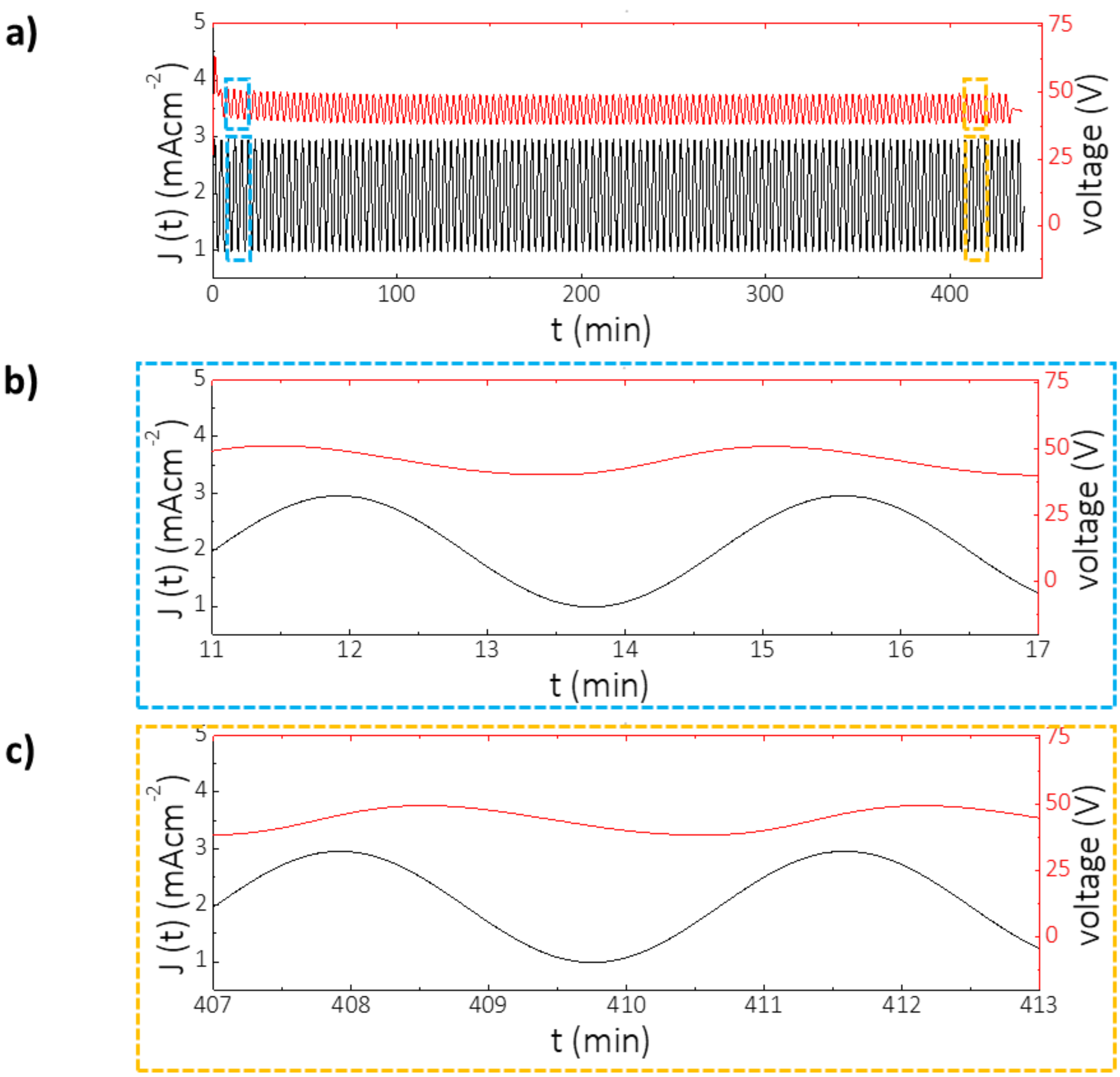

Figure S1. Fabrication of NAA–GIFs by sinusoidal pulse–like anodization. a) Representative full input sinusoidal current density profile (black solid line) and representative full output sinusoidal voltage profile (red solid line). The slight deviation of the voltage profile in the initial part towards higher voltage values is related to the uneven growth of the nanopores starting from a smooth surface. b) Magnified view (down left in blue dash line) of one period at the beginning of the electrochemical process with graphical definition of input anodization parameters: J_{max} – current density amplitude, $J_{average}$ – current density average, T – anodization period and the output parameters in voltage profile: $V_{average}$ – average voltage during the anodization process, V_{max} – output voltage amplitude. There is a time delay between input current density profile and voltage profile due to a slow current recovery process takes place out of equilibrium when input anodization current is altered; c) Magnified view of a representative input and output sinusoidal current density and voltage profile (down right in yellow dash line) with a graphical definition of parameters at the end of the electrochemical process of fabrication.

Table S1. Summary of the main structural and optical features of NAA–GIF–B s at different pore widening times.

t_{pw} (min)	d_p (nm)	d_{int} (nm)	λ_{PSB} (nm)	$FWHM_{PSB}$ (nm)
0	23 ± 4	93,1 ± 21,0	704 ± 1	21 ± 1
5	34 ± 4	109,1 ± 21,8	692 ± 1	25 ± 1
10	44 ± 5	124,1 ± 29,7	672 ± 1	29 ± 1
15	55 ± 5	110,3 ± 24,1	647 ± 1	36 ± 1

t_{pw} – pore widening time

d_p – pore diameter

d_{pwint} – interpore distance

λ_{PSB} – wavelength of the photonic stopband

$FWHM_{PSB}$ – full width at half maximum of the photonic stopband

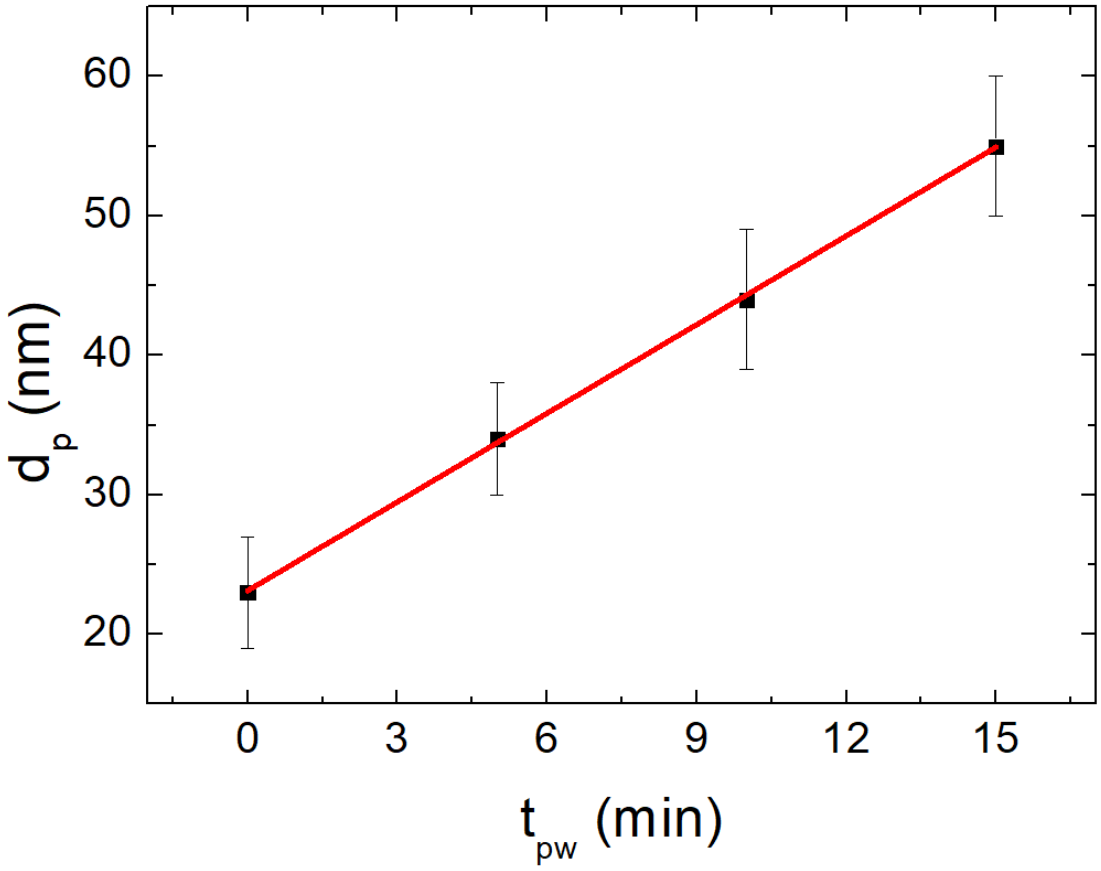

Figure S2. Linear correlation between pore diameter and pore widening time in NAA–GIF–B. Linear fitting line showing the dependence of pore diameter (d_p) with pore widening time (t_{pw}).

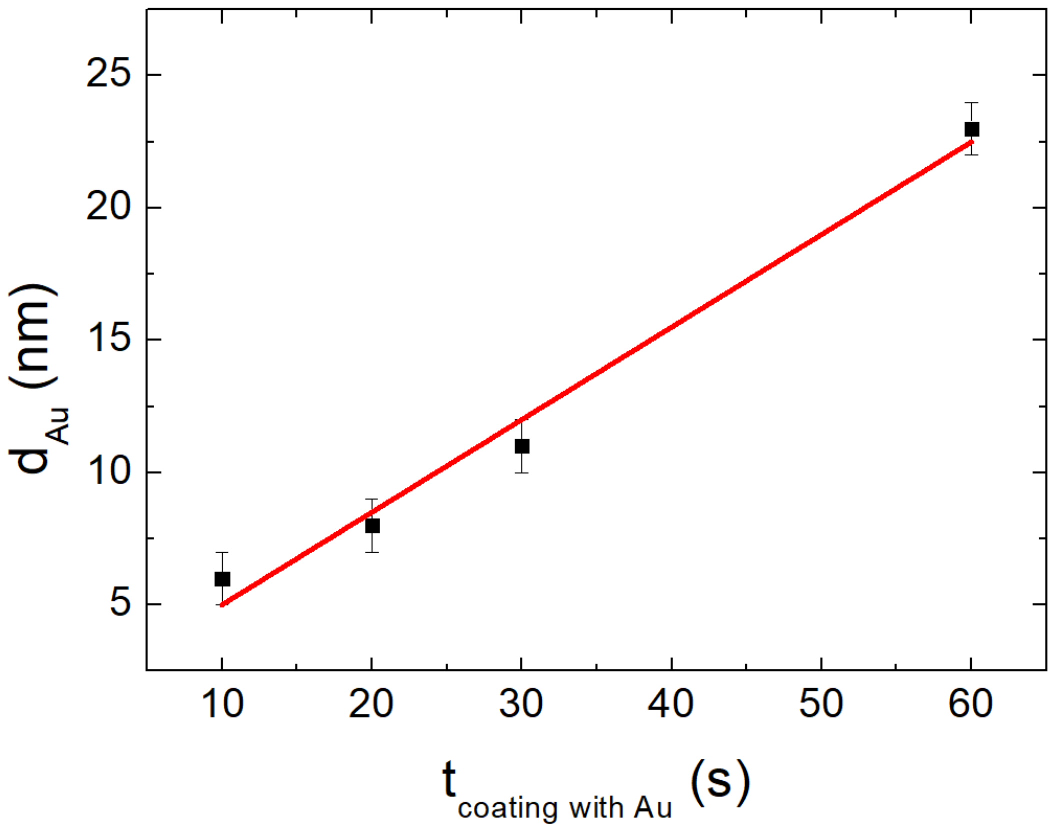

Figure S3. Calibration curve correlating porous gold coating layer thickness with deposition time in sample NAA–GIFs–B under the conditions of study (vacuum pressure of 5·10–5 mbar).

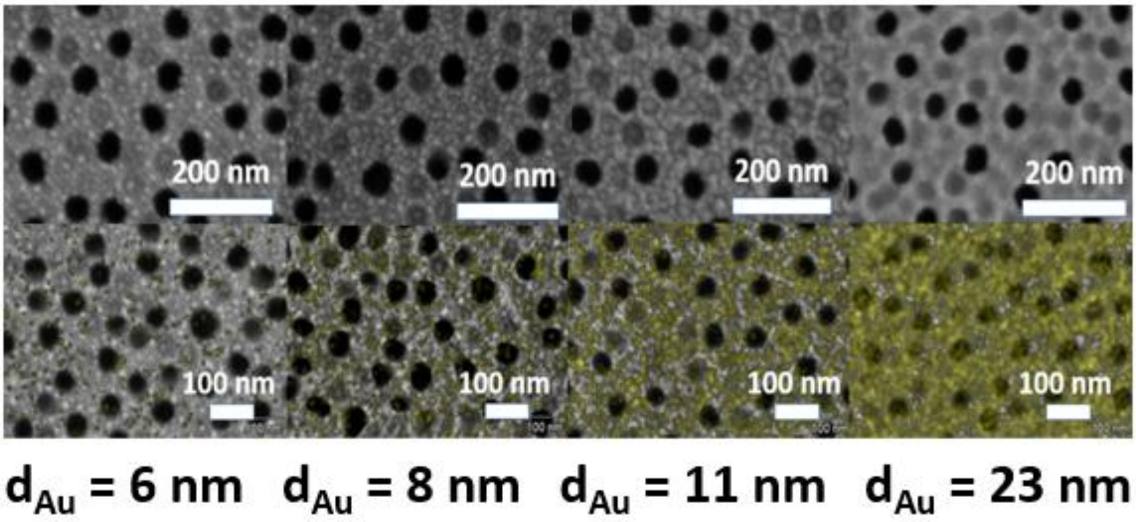

Figure S4: Elemental composition distribution map of gold deposited throughout the surface of a representative NAA–GIF–A after 15 min of pore widening. In the upper row, FEGSEM images for each thickness of gold sputtered on the NAA–GIF; in the lower row, in yellow, the gold sputtered on the NAA–GIF covers almost the entire top of the surface for each of the deposited thicknesses.

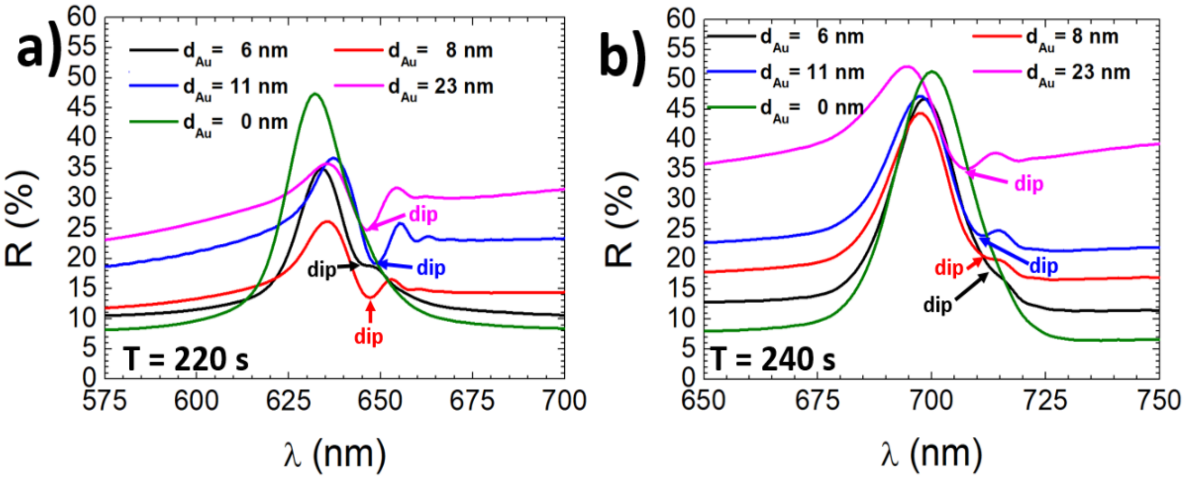

Figure S5. Reflection spectrum of as-produced Au-coated NAA–GIFs reveal an apparent dip within the PSB in the original spectrum. More intense dip in sample NAA–GIF–A represented in a) than sample NAA–GIF–B, represented in b). A qualitative enhancement in resolution of the Tamm plasmon resonance occurs when the porous gold coating layer thickness is increased. This enhancement is accompanied by an increase in baseline at thicker thicknesses.

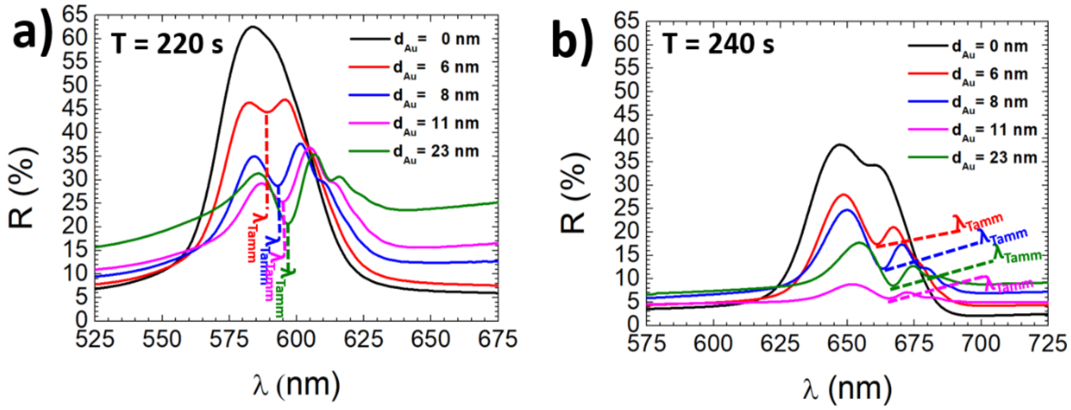

Figure S6. Reflection spectrum of Au-coated NAA–GIFs after 15 min of pore widening reveal we still have dip within the PSB in the original spectrum compared to **figure S3**. More intense dip in sample NAA–GIF–A represented in a) again compared to sample NAA–GIF–B, represented in b) but PSB is broader. Reflectance spectrum is better resolved in both NAA–GIF–A and NAA–GIF–B, but the resonance band λ_{Tamm} is more intense for NAA–GIF–A, which features a PSB located at shorter wavelengths.

Table S2. Summary of the main optical features of NAA-GIF for different d_{Au}. The dips corresponding to d = 6 and d = 8 nm have been indicated in an approximate area but cannot be determined graphically because they are barely resolved.

**NAA–GIF–A (low porosities)**			
d_{Au} (nm)	λ_{Tamm} (nm)	$FWHM_{Tamm}$ (nm)	Q_{Tamm}
6 ± 1	634 ± 1	10 ± 1	63 ± 1
8 ± 1	635 ± 1	13 ± 1	49 ± 1
11 ± 1	637 ± 1	15 ± 1	43 ± 1
23 ± 1	635 ± 1	16 ± 1	40 ± 1
**NAA–GIF–A (high porosities)**			
d_{Au} (nm)	λ_{Tamm} (nm)	$FWHM_{Tamm}$ (nm)	Q_{Tamm}
6 ± 1	589 ± 1	4 ± 1	147 ± 1
8 ± 1	593 ± 1	8 ± 1	74 ± 1
11 ± 1	595 ± 1	6 ± 1	99 ± 1
23 ± 1	597 ± 1	9 ± 1	66 ± 1
**NAA–GIF–B (low porosities)**			
d_{Au} (nm)	λ_{Tamm} (nm)	$FWHM_{Tamm}$ (nm)	Q_{Tamm}
6 ± 1	712 ± 1	--	--
8 ± 1	712 ± 1	--	--
11 ± 1	711 ± 1	3 ± 1	237 ± 1
23 ± 1	708 ± 1	5 ± 1	142 ± 1
**NAA–GIF–B (high porosities)**			
d_{Au} (nm)	λ_{Tamm} (nm)	$FWHM_{Tamm}$ (nm)	Q_{Tamm}
6 ± 1	661 ± 1	6 ± 1	110 ± 1
8 ± 1	663 ± 1	6 ± 1	111 ± 1
11 ± 1	666 ± 1	7 ± 1	95 ± 1
23 ± 1	667 ± 1	8 ± 1	83 ± 1

Table S3: NAA-GIF nanopores diameters after gold sputtering on the top surface.

$t_{COATING\ WITH\ Au}$ (s)	d_p (nm)
0	55 ± 5
10	50 ± 4
20	48 ± 5
30	50 ± 5
60	45 ± 5
